# Mechanism of Lakoochin A Inducing Apoptosis of A375.S2 Melanoma Cells through Mitochondrial ROS and MAPKs Pathway

**DOI:** 10.3390/ijms19092649

**Published:** 2018-09-06

**Authors:** Kuo-Ti Peng, Yao-Chang Chiang, Horng-Huey Ko, Pei-Ling Chi, Chia-Lan Tsai, Ming-I Ko, Ming-Hsueh Lee, Lee-Fen Hsu, Chiang-Wen Lee

**Affiliations:** 1 Department of Orthopaedic Surgery, Chang Gung Memorial Hospital, Puzi City, Chiayi County 61363, Taiwan; mr3497@adm.cgmh.org.tw; 2 College of Medicine, Chang Gung University, Guishan Dist., Taoyuan City 33303, Taiwan; 3 Department of Nursing, Division of Basic Medical Sciences, and Chronic Diseases and Health Promotion Research Center, Chang Gung University of Science and Technology, Puzi City, Chiayi County 61363, Taiwan; yaochang.chiang@gmail.com (Y.-C.C.); s041055@mail.cgust.edu.tw (M.-I.K.); 4 Department of Fragrance and Cosmetic Science, College of Pharmacy, Kaohsiung Medical University, Kaohsiung 80708, Taiwan; hhko@kmu.edu.tw; 5 Department of Medical Education and Research, Kaohsiung Veterans General Hospital, Zuoying Dist., Kaohsiung City 81362, Taiwan; chi542738@gmail.com; 6 Department of Pathology and Laboratory Medicine, Kaohsiung Veterans General Hospital, Zuoying Dist., Kaohsiung City 81362, Taiwan; 7 Department of Nursing, Division of Basic Medical Sciences, Chang Gung University of Science and Technology, Guishan Dist., Taoyuan City 33303, Taiwan; cltsai@mail.cgust.edu.tw; 8 Division of Neurosurgery, Department of Surgery, Chang Gung Memorial Hospital, Puzi City, Chiayi County 61363, Taiwan; ma2072@gmail.com; 9 Department of Respiratory Care, Chang Gung University of Science and Technology, Puzi City, Chiayi County 61363, Taiwan; 10 Research Center for Industry of Human Ecology and Research Center for Chinese Herbal Medicine, Chang Gung University of Science and Technology, Guishan Dist., Taoyuan City 33303, Taiwan; 11 Department of Rehabilitation, Chang Gung Memorial Hospital, Chang Gung Memorial Hospital, Puzi City, Chiayi County 61363, Taiwan

**Keywords:** lakoochin A, mitochondria, melanoma cells, MAPKs, pro-oxidation, apoptosis

## Abstract

Malignant melanoma is developed from pigment-containing cells, melanocytes, and primarily found on the skin. Malignant melanoma still has a high mortality rate, which may imply a lack of therapeutic agents. Lakoochin A, a compound isolated from *Artocarpus lakoocha* and *Artocarpus xanthocarpus*, has an inhibitory function of tyrosinase activity and melanin production, but the anti-cancer effects are still unclear. In the current study, the therapeutic effects of lakoochin A with their apoptosis functions and possible mechanisms were investigated on A375.S2 melanoma cells. Several methods were applied, including 3-(4,5-Dimethylthiazol-2-yl)-2,5- diphenyltetrazolium bromide (MTT), flow cytometry, and immunoblotting. Results suggest that lakoochin A attenuated the growth of A375.S2 melanoma cells through an apoptosis mechanism. Lakoochin A first increase the production of cellular and mitochondrial reactive oxygen species (ROSs); mitochondrial ROSs then promote mitogen-activated protein kinases (MAPKs) pathway activation and raise downstream apoptosis-related protein and caspase expression. This is the first study to demonstrate that lakoochin A, through ROS-MAPK, apoptosis-related proteins, caspases cascades, can induce melanoma cell apoptosis and may be a potential candidate compound for treating malignant melanoma.

## 1. Introduction

Melanoma is a common form of skin cancer, especially among Caucasians [1]. The malignant form of melanoma, called malignant melanoma, still has a poor prognosis. Several methods, including surgical excision, chemotherapy, radiotherapy, and immunotherapy, have been applied to treat malignant melanoma. However, these treatment methods do not effectively reduce the adverse effects or increase the long-term survival rate [2,3]. For this reason, investigating novel agents or methods to increase the efficacy and prognosis of malignant melanoma treatment are of clinical importance.

Lakoochin A is a stilbene derivative identified from *Artocarpus lakoocha* [4] and *Artocarpus xanthocarpus* [5]. Evidence suggests that *Artocarpus* species have inhibitory activities against tyrosinase [5,6], pancreatic lipase [7], antibacterial (such as Mycobacterium tuberculosis), and antiviral activity (such as herpes simplex virus and human immunodeficiency virus) [8,9]. Furthermore, studies have also suggested that extractions from *Artocarpus* sp. show anti-cancer properties for melanoma [10], hepatocellular carcinoma [11], gastric carcinoma [12], and colorectal carcinoma [13]. The functions of lakoochin A have been reported to inhibit α-glucosidase activity [14], melanin biosynthesis [5], antimycobacterial activity against *Mycobacterium tuberculosis*, and cytotoxicity against breast cancer and nasopharyngeal carcinoma cell lines [4]. However, the details of lakoochin A on its anti-cancer function and related mechanisms are still unclear and require further investigation.

The roles of reactive oxygen species (ROSs) have been associated with anti-cancer function for some flavonoids from *Artocarpus* sp. [10,15,16]. It is well known that reactive oxygen species (ROSs) are a double-edged sword in terms of physiological and pathological organism functions [17,18,19]. For example, in physiological conditions, ROSs play important roles in phagocytosis, cell signaling, and homeostasis. Subsequently, reactive species could be eliminated by the scavenging system of normal cells [20,21]. However, under oxidative stress conditions, ROSs accumulate in higher concentrations and oxidize cellular lipids, proteins, and DNA. Finally, these ROSs cause aggravation and exacerbation of several clinical diseases and phenomena, such as inflammation, neurodegeneration, aging, cancer, and cardiovascular disease [21,22,23,24,25]. Additionally, some anti-cancer agents, isolated from traditional Chinese herbal medicine, such as paclitaxel [26], resveratrol [27], and curcumin [28], can increase ROS production to inhibit cancer growth, activate the mitogen-activated protein kinase (MAPK) pathway, and increase expression of apoptosis-related proteins. In this study, the role that lakoochin A plays in A375.S2 melanoma cell proliferation and apoptosis were investigated. The underlying mechanisms were also evaluated, including the ROSs, MAPK pathways, and their downstream signaling.

## 2. Results

### 2.1. Lakoochin A Inhibits Proliferation and Viability of A375.S2 Melanoma Cells

**Figure 1:**
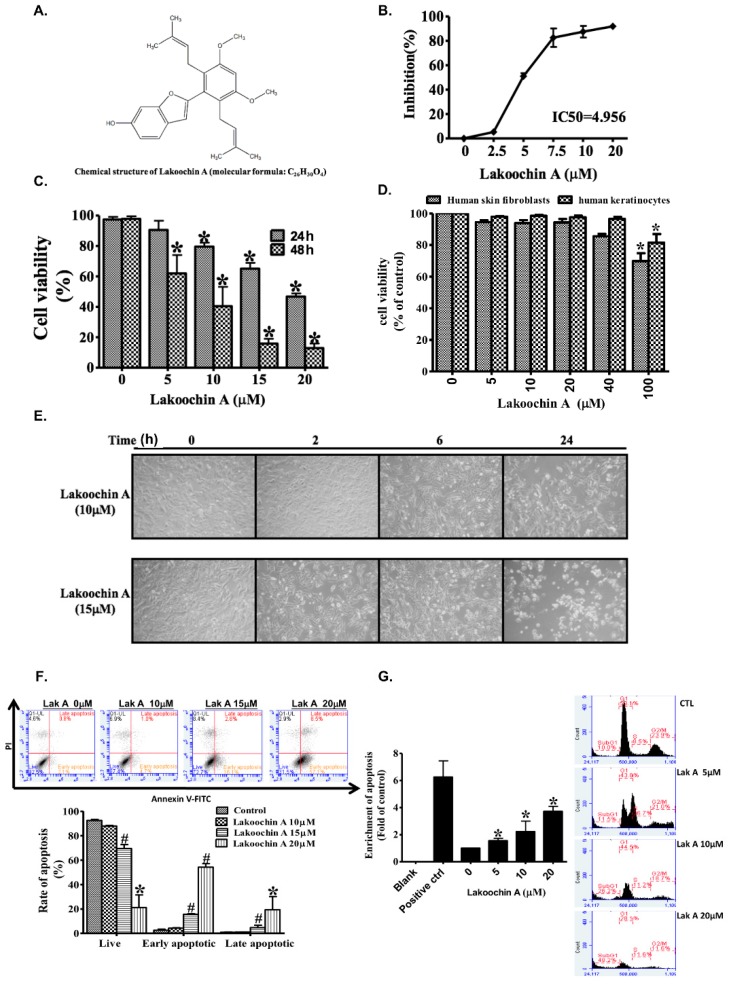
(**A**) The chemical structure of lakoochin A. (**B**) The inhibitory effect of lakoochin A on A375.S2 cell proliferation, as determined by the SRB assay at 24 h. (**C**) Dose and time effects of lakoochin A on A375.S2 cell viability, as determined by the 3-(4,5-Dimethylthiazol-2-yl)-2,5- diphenyltetrazolium bromide (MTT) assay at 24 and 48 h. (**D**) The effects of lakoochin A on human skin fibroblast and keratinocytes as determined by the MTT assay at 24 h. The cell apoptosis effects of lakoochin A on A375.S2 cells, as (**E**) presented by the morphology and (**F**) determined by flow cytometry with AnnexinV-Fluorescein isothiocyanate (FITC) and propidium iodide staining at 24 h. The right lower quadrant indicates early apoptosis. (**G**) Effects of lakoochin A on cell apoptosis (**left panel**) and sub-G1 cell cycle arrest (**right panel**) were determined by DNA fragmentation assay and flow cytometry, with propidium iodide stainingon A375.S2 cells at 24 h, respectively. Results (**B**–**G**) expressed as mean ± S.E.M. from three individual experiments. * *p* < 0.05 and # *p* < 0.01 compared to the control group.

Cell proliferation was assayed by using the Sulforhodamine B (SRB) assay. Results showed that treatment with lakoochin A (2.5–20 μM, dissolved in dimethyl sulfoxide (DMSO) on A375.S2 melanoma cells for 24 h could inhibit cell proliferation in a concentration-dependent manner and with a half maximal inhibitory concentration (IC50) value of 4.956 μM (Figure 1B). The MTT assay suggested that lakoochin A treatment for 24 or 48 h reduced the cell viability in a concentration-dependent manner (0–20 μM, Figure 1C). Additionally, as shown in Figure 1D, lakoochin A did not significantly change the cell viability of human skin fibroblasts and keratinocytes, until high doses (100 μM) were administered.

### 2.2. Lakoochin A Promotes Apoptosis and Cell Cycle Arrest in A375.S2 Melanoma Cells

Staining was used to test whether lakoochin A has an apoptosis function on A375.S2 cells, cell morphology and flow cytometry with AnnexinV-FITC and propidium iodide. As shown in Figure 1E, lakoochin A (10 and 15 μM) promoted apoptosis in a concentration- and time-dependent manner on A375.S2 cells. As shown in Figure 1F, the percentage of early apoptosis of cells after lakoochin A treatment for 24 h was 2.1% (0 μM), 4.7% (10 μM), 16.1% (15 μM), and 57.1% (20 μM). Treatment also led to a concentration-dependent increase in DNA fragmentation (Figure 1G, left panel). Furthermore, treatment with lakoochin A resulted in an increase in the percentage of cells being arrested in the sub-G1 phase (Figure 1G, right panel). The percentage of sub-G1 phase was observed as 10.0% (0 μM), 11.5% (5 μM), 26.2% (10 μM), and 48.2% (20 μM) in cells after lakoochin A treatment for 24 h.

### 2.3. Lakoochin A Increases Apoptosis of A375.S2 Cells through the Mitochondrial Pathway

**Figure 2:**
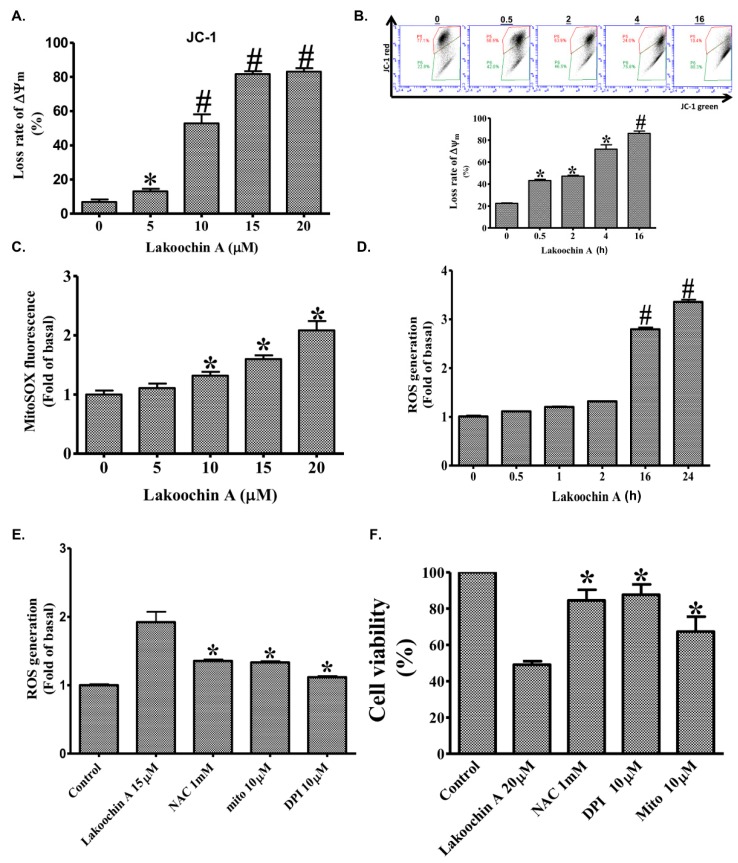
(**A**) The dose effect of lakoochin A at 24 h on the mitochondrial membrane potential (∆Ψm) of A375.S2 cells, as determined by flow cytometry staining with JC-1. (**B**) The time effects of lakoochin A on the ∆Ψm of A375.S2 cells pre-labeled with 5,5′,6,6′-Tetrachloro-1,1′,3,3′-tetraethylbenzimidazolylcarbocyanineiodide (JC-1) (10 μg/mL) for the indicated times (0.5–16 h). (**C**) Effect of lakoochin A on mitochondrial reactive oxygen species (ROS) production (determined by flow cytometry after staining with MitoSOX Red indicator) in A375.S2 cells. (**D**) Effect of lakoochin A on cellular ROS production (determined by flow cytometry after staining with H2DCFDA reagent). (**E**) The cellular ROS production of several antioxidants and lakoochin A, determined by flow cytometry staining with H2DCFDA reagent. The A375.S2 cells were pretreated for 1 h with mitochondria-targeted antioxidant (MitoTEMPOL), antioxidant (NAC), or nicotinamide adenine dinucleotide phosphate (NADPH) oxidase inhibitor (DPI) and then treated with lakoochin A for 4 h. (**F**) Effect of MitoTEMPOL, NAC, and DPI on lakoochin A-induced A375.S2 cell apoptosis (determined by MTT assay). The data were collected from at least three individual experiments and expressed as mean ± S.E.M. * *p* < 0.05, # *p* < 0.01 compared to the control group.

The 5,5′,6,6′-Tetrachloro-1,1′,3,3′-tetraethylbenzimidazolylcarbocyanineiodide (JC-1) assay showed that the treatment of A375.S2 cells with lakoochin A (2.5–20 μM) for 24 h decreased mitochondrial membrane potential in a concentration- and time-dependent manner (Figure 2A,B). This result indicates that lakoochin A raised apoptosis in A375.S2 cells, affecting the mitochondrial functions.

### 2.4. Lakoochin A Induces Cellular and Mitochondrial ROS Production in A375.S2 Cells

Figure 2C shows the dose-dependent manner of lakoochin A-induced mitochondrial ROS production (determined by MitoSOX). According to the results of the whole cell ROS generation (determined by H2DCFDA), lakoochin A (10 µM) induced cellular ROS production in a time-dependent manner in A375.S2 cells (Figure 2D). Furthermore, several inhibitors were used to identify the source of ROS production in cells. As shown in Figure 2E, lakoochin A (15 µM)-induced cellular ROS production (determined using the H2DCFDA assay) could be attenuated by a general antioxidant (NAC, 2 mM), a mitochondria-targeted antioxidant (MitoTEMPOL, 10 µM), and a NADPH oxidase inhibitor (DPI, 2 mM). Similarly, lakoochin A (20 µM) caused a decrease in the viability of A375.S2 cells which could be reversed by NAC, MitoTEMPOL, and DPI. This indicates that lakoochin A-induced cell death is required for ROS production.

### 2.5. Lakoochin A Induces Mitochondrial ROS Generation to Activate the MAPK Pathway in A375.S2 Cells

**Figure 3:**
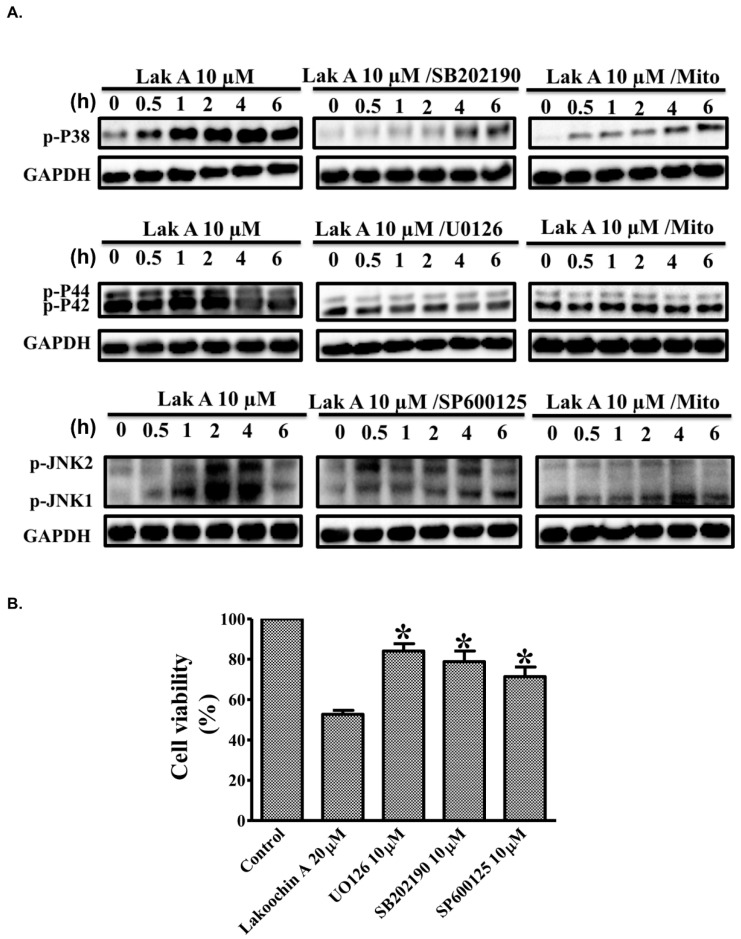
(**A**) Effects of different time course of lakoochin A, with or without being pre-treated for 1 h with a p38 inhibitor (SB202190), MEK1/ERK inhibitor (U0126), JNK inhibitor (SP600125), and mitochondria-targeted antioxidant (MitoTEMPOL), on expressions of the phosphorylation status of p38, ERK (p44/p42), and JNK in A375.S2 cells. Glyceraldehyde 3-phosphate dehydrogenase (GAPDH) was used as a loading control. Blots were representative of three independent experiments. (**B**) Effect of U1026, SP600125, and MitoTEMPOL on lakoochin A-induced A375.S2 cell apoptosis (determined by MTT assay). The data were collected from at least three individual experiments and expressed as mean ± S.E.M. * *p* < 0.05 as compared to the control group.

To characterize the effects of lakoochin A on MAPK pathways, Western blotting was conducted in A375.S2 (Figure 3A). As shown, lakoochin A (10 µM) induced phosphorylation of p38, extracellular signal–regulated kinases (ERK, p44/p42), and c-Jun N-terminal kinases (JNK), in a time-dependent manner, in A375.S2 cells (from 0 to 6 h). On the other hand, these phosphorylation effects could be suppressed by pre-treating specific inhibitors (SB-202190, U-0126, and SP600125, respectively) and MitoTEMPOL (mitochondria-targeted antioxidant, 10 µM) for 1 h. Furthermore, lakoochin A (20 µM) caused decreases in the viability of A375.S2 cells which could be reversed by MAPKs inhibitors, namely SB-202190, U-0126, and SP600125. The results indicate that lakoochin A-induced MAPK pathways activation can be generated by mitochondrial ROSs in A375.S2 melanoma cells.

### 2.6. Lakoochin A Induces Apoptosis-Related Protein Expression in A375.S2 Cells via Mitochondrial ROS Generation

**Figure 4:**
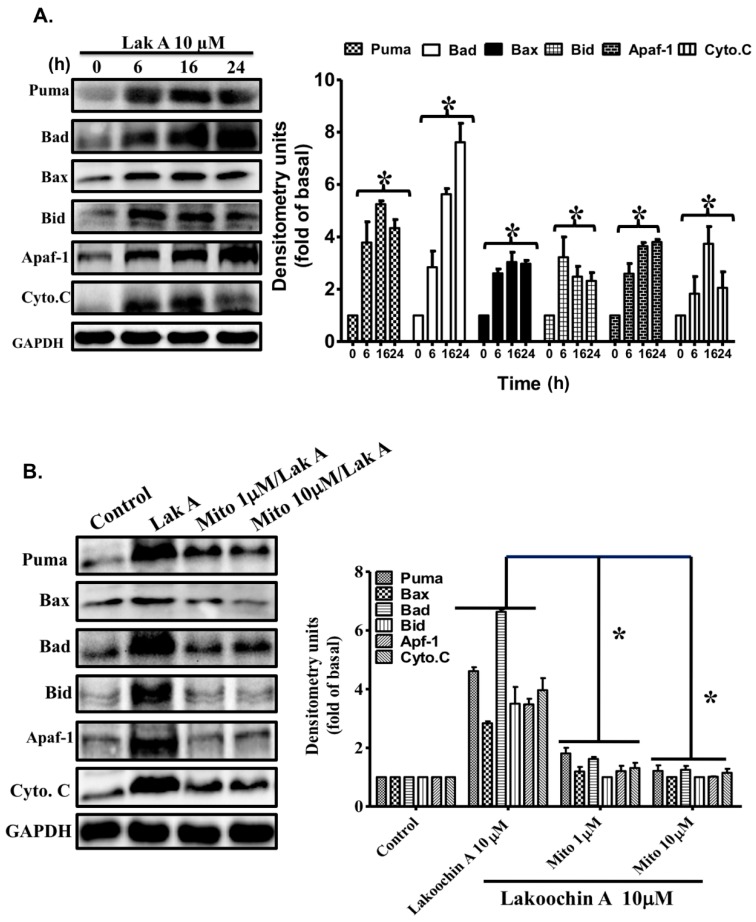
(**A**) Effects of lakoochin A on the expression levels of apoptosis-related proteins Puma, Bax, Bad, Bid, Apaf-1, and cytochrome c in A375.S2 cells, over various time periods (0–24 h) (**B**) The cells were pre-treated with 1 and 10 μM MitoTEMPOL for 1 h followed by treatment of lakoochin A for 16 h. Expression of apoptosis-related proteins was determined by Western blotting. GAPDH was used for a loading control. The intensity of the bands was quantified by densitometry, and the data were collected from three individual experiments and expressed as mean ± S.E.M. * *p* < 0.05, compared to the control group.

According to the Western blotting results, lakoochin A (10 µM) can increase the apoptosis-related protein expression, such as Puma, Bax, Bad, Bid, Apaf-1, and cytochrome c, in a time-dependent manner (from 0 to 24 h) (Figure 4A). Furthermore, the mitochondria-targeted antioxidant (MitoTEMPOL with 1 or 10 µM) was pre-treated to confirm the role of mitochondrial ROSs. As shown in Figure 4B, 10 µM lakoochin A-induced expression of Puma, Bax, Bad, Bid, Apaf-1, and cytochrome c can be suppressed by treatment with MitoTEMPOL in A375.S2. These results suggest that lakoochin A promotes apoptosis-related protein expression in A375.S2 cells dependent on mitochondrial ROS production.

### 2.7. Lakoochin A Induces the Activation of Caspase-3, Caspase-7, and Caspase-9 in A375.S2 Cells

**Figure 5:**
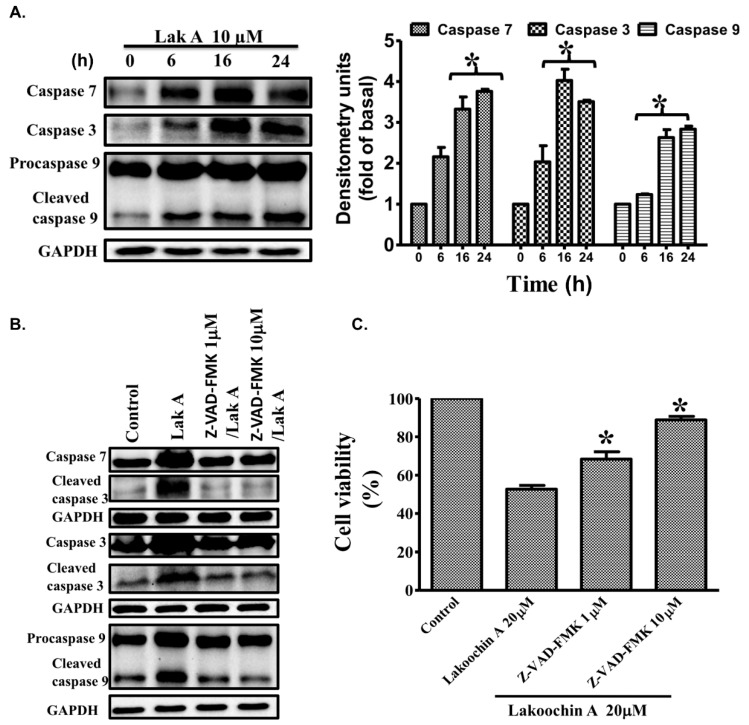
(**A**) Effects of lakoochin A (10 μM) on the expression status of caspase-7, caspase-3, and caspase-9 in A375.S2 cells in a serial time-period (0–24 h). (**B**) Effects of caspase inhibitor carbobenzoxy-valyl-alanyl-aspartyl-[O-methyl]-fluoromethylketone (Z-VAD-FMK) on lakoochin A-induced increases in caspase-3, -7, and -9. Cells were pre-treated for 1 h with Z-VAD-FMK (1 or 10 μM) followed by treatment with lakoochin A for 16 h. The levels of caspase-3, -7, and -9 were evaluated by immunoblotting. (**C**) Effect of Z-VAD-FMK (1 or 10 μM) on lakoochin A-induced A375.S2 cell apoptosis (determined by MTT assay). The data were collected from at least three individual experiments and expressed as mean ± S.E.M. * *p* < 0.05, as compared to the control group.

Caspases play a critical role in cell inflammation and death [29]. Caspase-3, caspase-7, and caspase-9 in particular, play important roles during apoptosis [30]. Hence, the effects of lakoochin A on caspase-7, caspase-3, and caspase-9 were conducted. As shown in Figure 5A, treatment of the A375.S2 cells with lakoochin A (10 µM) increased the expression of caspase-7, caspase-3, and caspase-9 in a time-dependent manner (from 0 to 24 h). Additionally, the activation of caspase-7, caspase-3, and caspase-9 in A375.S2 cells can be significantly suppressed by pre-treating with 1 and 10 µM caspase inhibitor Z-VAD-FMK (Figure 5B). Like the caspase immunoblotting results, the decrease effect of lakoochin A (20 µM) on cell viability of A375.S2 cells can also be blocked by the caspase inhibitor carbobenzoxy-valyl-alanyl-aspartyl-[O-methyl]-fluoromethylketone (Z-VAD-FMK). This result suggests that lakoochin A induced apoptosis of A375.S2 cells, which mediated caspase pathways.

## 3. Discussion

In the current study, we first demonstrate that lakoochin A has anti-melanoma cancer cell activity linked to ROS generation and MAPK pathways. Lakoochin A treatment in A375.S2 cells results in apoptosis via an increase in ROS formation, while pre-treatment of NAC, DPI, and MitoTEMPOL appear to reduce ROS generation from cellular and mitochondria. In addition, the expressions of phosphorylation type of p38, ERK (p44/p42), and JNK can be suppressed by SB-202190, U-0126, SP600125, and MitoTEMPOL respectively, indicating that MAPK pathways are down-stream pathways of ROSs. Furthermore, inhibiting mitochondrial ROS formation also reduces expressions of various apoptosis-related proteins induced by lakoochin A. This may imply that lakoochin A-induced apoptosis to A375.S2 cell requests the ROS production of mitochondria. In an attempt to characterize the possible cytotoxic mechanisms of lakoochin A, we analyzed the levels of apoptosis-related proteins, and caspase expression. Results suggest that lakoochin A promotes an increase of apoptosis-related protein via mitochondrial ROS formation. Moreover, caspase-3, -7 and -9 activities and expressions, that were increased by lakoochin A, can be suppressed with treatment of MitoTEMPOL, a mitochondria-targeted antioxidant.

Malignant melanoma, a kind of transformation and uncontrolled growth of melanocytes, is an aggressive and most malignant form of skin cancer [2]. Although several medicines have been developed to treat malignant melanoma the incidence rates of malignant melanoma are still increasing, especially in the Caucasian population [1]. Malignant melanoma causes a higher mortality rate. Svedman et al. [31] suggests that, among patients with melanoma stage IV, there is only a 9–28%, five-year survival rate. These findings may imply that there is still a lack of therapeutic agents. In clinical practice, melanoma may be initially observed anywhere on the skin, as pigmented or non-pigmented papules, plaques, or nodules, particularly in sun-exposed areas. Malignant melanocytes rapidly metastasize to lymph nodes and pass to other visceral organs [3]. Several methods, such as surgery, radiotherapy, chemotherapy, immunotherapy, and biological agents, have been applied to treat melanoma [32]. Unfortunately, there is still no method for increasing long-term survival rate for malignant melanoma patients. Therefore, the task of identifying novel compounds with potential therapeutic effects for treating melanoma is urgent.

Recently, various bioactive compounds derived from natural sources have been demonstrated as candidates for anti-cancer medicines. Evidence suggests that compounds from *Artocarpus* species have cytotoxic functions for treating cancer. Lakoochin A (Figure 1A) contains stilbene derivatives isolated from *Artocarpus lakoocha* [4] and *Artocarpus xanthocarpus* [5]. Previous studies suggest lakoochin A has functions on the inhibition of α-glucosidase activity [14], melanin biosynthesis [5], antimycobacterial activity and cytotoxicity for breast cancer, and nasopharyngeal carcinoma cell lines [4]. This study is the first to demonstrate that lakoochin A has anti-melanoma effects.

It is well known that cell apoptosis may be a result of exposure with an increase in oxidative stress. ROSs produced from cells may have two main sources, namely NADPH oxidase and mitochondria. This mechanism involves anti-cancer growth. For example, Beberok et al. [33] suggested that lomefloxacin, a kind of fluoroquinolone, can induce ROSs and reduce topoisomerase II to cause apoptosis of COLO829 melanoma cells. Similar mechanisms were also found in the current study. Lakoochin A can increase cellular and mitochondrial ROS formation in A375.S2 cells and be suppressed by pre-treating with NAC (cellular antioxidant), DPI (NADPH oxidase inhibitor), and MitoTEMPOL (mitochondria antioxidant). However, the major source of ROSs, generated by lakoochin A, requires further investigation. Various signal transduction pathways can be activated upon oxidative stress formation in cells. It has been demonstrated that the MAPK signaling pathway plays an important regulation role in human diseases, including cancer growth and metastasis [34,35]. ERK1/2 (p44/p42), JNK, and p38 are the three main components in human cells. They have different functions: ERK1/2 mainly mediates in cell proliferation, survival, migration, and invasion, while JNK and p38 are involved in cell apoptosis [36,37]. Previous studies suggest that resveratrol, a kind of stilbene [38], presents antitumor activity with respect to melanoma cells associated with the suppression of telomerase [39] and attenuates the Akt/PKB activity [40], via the p53 pathway. The activation of caspase-9 and caspase-3, works to upregulate Bcl-2-associated X protein and B-cell lymphoma 2 expressions [41], and the activation of ERK1/2, but not p38 or JNK [42]. Our results show that lakoochin A can activate caspase-9, -7 and -3, as well as the phosphorylation of ERK1/2 (p44/p42), p38, and JNK. They seem to present differently with resveratrol, which implies that the stilbene-induced apoptosis of cancer cells may occur via multiple pathways. Furthermore, the phosphorylation of MAPK signaling can be suppressed by pre-treating cells with specific MAPK inhibitors (SB-202190, U-0126, and SP600125) and by a mitochondria-targeted antioxidant (MitoTEMPOL). These findings strongly suggest that the lakoochin A-induced activation of MAPK signaling in A375.S2 melanoma cells, occurs via the mediation of mitochondrial ROS production. These results are consistent with previous studies where mitochondrial ROS generation promoted MAPK activation and led to apoptosis [15,43].

Two distinct pathways, the death receptor and mitochondrial (death receptor-independent) pathways, can bring about apoptosis [44,45]. Cellular stress responses may lead pro-apoptotic proteins of the Bcl-2 family (Puma, Bax, Bad, and Bid) to translocate from cytosol into mitochondria through the mitochondrial pathway [46,47]. This results in a rise in the mitochondrial permeability and activation of caspases, via releases of cytochrome c from the mitochondria [48,49]. Cytochrome c coordinates with Apaf-1 and procaspase-9 to form the apoptosome [50], which regulates the caspase 9 homo- and heterodimer [51], and subsequently activates caspase-9 and caspase-3/7, causing apoptosis [52]. This was also observed in the current study. Lakoochin A increased the expression of the pro-apoptotic Bcl-2 family (Puma, Bax, Bad, and Bid), Apaf-1, and cytochrome c, which can be attenuated by pre-treatment with MitoTEMPOL in A375.S2 melanoma cells. Additionally, lakoochin A can decrease the mitochondrial membrane potential and increase the activity of caspase-9, -7 and -3. This indicates that lakoochin A induces apoptosis for melanoma cells via the mitochondrial pathway.

In conclusion, our study is the first to demonstrate that lakoochin A causes cytotoxicity for A375.S2 melanoma cells. It regulates mitochondrial ROS production, increases pro-apoptotic proteins of the Bcl-2 family (Puma, Bax, Bad, Bid) expression, MAPK (p38/ERK/JNK) signaling activation, and the activation of caspases. Therefore, lakoochin A may be considered as a potential agent for treating malignant melanoma.

## 4. Materials and Methods

### 4.1. Materials

A human melanoma cell line (A375.S2) was provided by Feng-Lin Yen (Kaohsiung Medical University). Dulbecco’s Modified Eagle Medium/Nutrient Mixture F-12 (DMEM/F-12) was obtained from GIBCO (Grand Island, NY, USA) and supplemented with 10% fetal bovine serum (FBS) (Hazelton Research Products, Denver, PA, USA). Antibodies for Western blotting (phospho-p38, phospho-ERK, and phospho-JNK) were obtained from Cell Signaling Technology (Danvers, MA, USA). Puma, Bax, Bad, Bid, Apaf-1, cytochrome C, caspase-3, caspase-7, and caspase-9 were obtained from Santa Cruz Biotechnology (Santa Cruz, CA, USA). The GAPDH antibody was obtained from Biogenesis (Bournemouth, UK). *N*-acetylcysteine (NAC), apocynin (APO), U-0126, SB-202190, and SP600125 were procured from Biomol (Plymouth Meeting, PA, USA). MitoSOX Red mitochondrial superoxide indicator was purchased from Molecular Probes (Eugene, OR, USA). Lakoochin A was provided by co-author Horng-Huey Ko (Kaohsiung Medical University), and isolated from the methanolic extract of *A. xanthocarpus* as described previously in [5,6]. Its purity was determined (>97%) using high-performance liquid chromatography (HPLC) analysis. The lakoochin A was powdered and stored in a black bottle at −20 °C, and dissolved in DMSO before the experiments were performed. Each control group was treated equivalent to DMSO volume of their tested groups.

### 4.2. Cell Culture

A human melanoma cell line (A375.S2) was grown in culture utilizing Dulbecco’s Modified Eagle Medium/Nutrient Mixture F-12 (DMEM/F-12, Gibico, Grand Island, NY, USA) supplemented with 10% FBS (Hazelton Research Products, Denver, PA, USA). Cells were maintained at 37 °C in a humidified atmosphere of 5% CO_2_/95% air.

### 4.3. Cell Proliferation Assay

Cell proliferation was performed by the sulforhodamine B (SRB) assay in accordance with a previous study [15]. Briefly, A375.S2 cells were treated with lakoochin A (isolated from methanolic extract of *Artocarpus xanthocarpus* as described in a previous study [5]) for 24 h and subsequently fixed in 10% trichloroacetic acid (TCA). The cells were then stained with SRB (Sigma-Aldrich, St. Louis, MO, USA) and washed with acetic acid (1%). Cell-bound SRB dye was dissolved with Tris-base (10 mM), and the absorbance was measured with a spectrometer at a wavelength of 515 nm.

### 4.4. Cell Viability Assay

The cell viability of A375.S2 was determined using the 3-(4,5-dimethylthiazol-2-yl)-2,5-diphenyltetrazolium bromide (MTT) assay (Sigma-Aldrich). First, cells were plated onto 96-well plates and incubated overnight before testing. The next morning, different concentrations of lakoochin A cells were added for 24 or 48 h. After MTT solution incubation at 37 °C for 1 h, the plates were read at 550 nm.

### 4.5. Cell Apoptosis Measurement

The cell apoptosis was measured by flow cytometry with the Annexin V-FITC/propidium iodide assay kit (Thermo Fisher Scientific, Waltham, MA, USA) and DNA fragmentation assay. Different concentrations of lakoochin A (10, 15, and 20 μM) were added to A375.S2 cells for 24 h, and these cells were then stained with Annexin V-FITC and propidium iodide, according to the manual, and subjected to flow cytometry (Accuri C6, BD Biosciences, San Jose, CA, USA).

### 4.6. Cell Cycle Analysis

Following treatment with varying concentrations of lakoochin A (5, 10, and 20 µM) for 24 h, A375.S2 cells were stained with RNAase and propidium iodide, then subjected to an Accuri C6 Flow Cytometer (BD Biosciences, San Jose, CA, USA). In the DNA fragmentation assay, A375.S2 cells were treated with different concentrations of lakoochin A (5, 10, and 20 μM) for 24 h, and the degree of DNA fragmentation was measured using the Cell Death Detection ELISA kit (Roche Diagnostics, Basel, Switzerland) and read by the ELISA reader.

### 4.7. Assessment of Mitochondrial Membrane Potential

A375.S2 cells were seeded onto 96-well plates (dose) or 12-well plates (time) and then incubated overnight. Cells were then treated with lakoochin A at varying concentrations for 24 h or 10 μM with different time intervals. Cells were stained with 5,5′,6,6′-Tetrachloro-1,1′,3,3′-tetraethylbenzimidazolylcarbocyanineiodide (JC-1; Sigma-Aldrich). The cells were incubated at 37 °C for 30 min and analyzed using a flow cytometer at 590 nm and 520 nm (for red and green fluorescence, respectively). The ratio of fluorescence (590/520 nm) reflected the change in mitochondrial membrane potential.

### 4.8. Determination of Cellular and Mitochondrial ROS Production

Cellular ROS production was measured using the 2′,7′-dichlorodihydrofluorescein diacetate (H2DCFDA) assay. A375.S2 cells were seeded onto 12-well plates and incubated overnight. Cells were treated with different concentrations of lakoochin A, with or without inhibitors for different amounts of time, and then stained with H2DCFDA Reagent (Thermo Fisher Scientific, USA). The fluorescence intensity of the cells was assessed using flow cytometry (excitation wavelength, 488 nm; emission wavelength, 530 nm). Furthermore, MitoSOX^TM^, a Red Mitochondrial Superoxide Indicator assay was used for evaluating the production of the mitochondrial ROSs. In accordance with the treatment schedule described above, after lakoochin A treatment, cells were stained with MitoSOX Red mitochondrial superoxide indicator (Molecular Probes, Eugene, OR, USA) and measured via flow cytometry (excitation wavelength, 488 nm; emission wavelength, 585 nm). The inhibitors, *N*- acetylcysteine (NAC) and diphenylene iodonium (DPI), were purchased from Biomol (Plymouth Meeting, PA, USA); MitoTEMPOL was purchased from Cayman Chemical (Ann Arbor, MI, USA).

### 4.9. Immunoblotting Analysis for MAPKs, Apoptosis-Related Proteins, and Caspases

A375.S2 cells were seeded onto 12-well plates and incubated overnight. Cells were treated with 10 μM lakoochin A (with or without inhibitors) for different amounts of time. Following treatment, the cells were lysed in a lysis buffer and subjected to protein extraction. Afterward, equal amounts of the protein were separated by SDS-polyacrylamide gel electrophoresis (10–12.5% polyacrylamide) and transferred onto nitrocellulose membranes. Samples were incubated overnight with different primary antibodies to measure the levels of the targets (phospho-p38, phospho-ERK, and phospho-JNK (Cell Signaling Technology, Danvers, MA, USA), Puma, Bax, Bad, Bid, Apaf-1, cytochrome C, caspase-3, caspase-7, and caspase-9 (Santa Cruz Biotechnology, Santa Cruz, CA, USA), and GAPDH (Biogenesis, Bournemouth, UK)). Next, secondary antibodies conjugated with horseradish peroxidase were added, and immunoreactivity was assessed using the enhanced chemiluminescence (ECL) detection system. The signals were detected on a ChemiDoc^TM^ XRS+ image system (Bio-Rad Laboratories, Hercules, CA, USA). The inhibitors, U-0126, SB-202190 and SP600125, were purchased from Biomol (Plymouth Meeting, PA, USA). The immunoblotting data were collected from at least three replicates with a similar pattern. The value was calculated from those figures, then a closer figure pattern was selected to present the statistical values.

### 4.10. Statistical Analysis

All data were analyzed using GraphPad Prism software (v4, GraphPad, San Diego, CA, USA). Results were expressed as mean ± SEM. Continuous data were analyzed by one-way or two-way ANOVA followed by post-hoc Tukey’s multiple comparison (multiple groups). A *p* value < 0.05 was considered significant.

## Abbreviations

Apaf-1	apoptotic protease activating factor 1
Bad	Bcl-2-associated death promoter
Bax	BH3-interacting domain death agonist (Bid), BCL2-associated X protein
DPI	diphenylene iodonium
ERK	extracellular regulated protein kinase
JNK	c-Jun N-terminal kinase
MAPK	mitogen-activated protein kinases
NAC	*N*-acetylcysteine
ROS	reactive oxygen species
Puma	p53 upregulated modulator of apoptosis
